# Syphilis in during pregnancy: association of maternal and perinatal characteristics in a region of southern Brazil^1^

**DOI:** 10.1590/1518-8345.2305.3019

**Published:** 2018-08-09

**Authors:** Camila Padovani, Rosana Rosseto de Oliveira, Sandra Marisa Pelloso

**Affiliations:** 2 MSc, RN, Hospital e Maternidade Santa Rita, Maringá, PR, Brazil.; 3 Post-doctoral fellow, Universidade Estadual de Maringá, Maringá, PR, Brazil. Scholarship holder at Coordenação de Aperfeiçoamento de Pessoal de Nível Superior (CAPES), Brazil.; 4 PhD, Full Professor, Departamento de Enfermagem, Universidade Estadual de Maringá, Maringá, PR, Brazil.

**Keywords:** Syphilis, Pregnancy, Risk Factors, Syphilis, Congenital, Sexually Transmitted Diseases, Maternal and Child Health

## Abstract

**Objective:**

To analyze the prevalence of syphilis in during pregnancy and its association with socioeconomic characteristics, reproductive history, prenatal and labor care, and newborn characteristics.

**Method:**

A retrospective, cross-sectional study based on gestational and congenital syphilis reports. A (records) linkage was performed in the Brazilian databases: “Information System for Notifiable Diseases” *(Sistema de Informação de Agravos de Notificação* - *SINAN*); “Live Births Information System” (*Sistema de Informação sobre Nascidos Vivos* - *SINASC*); and “Mortality Information System” (*Sistema de Informação sobre Mortalidade* - *SIM*).

**Results:**

The prevalence of gestational syphilis was 0.57%. The following associations of syphilis in pregnancy were found: non-white skin color/ethnicity (PR=4.6, CI=3.62-5.76); low educational level (PR=15.4; CI=12.60-18.86); and absence of prenatal care (PR=7.4, CI=3.68-14.9). The perinatal outcomes associated with gestational syphilis were prematurity (PR=1.6 CI=1.17-2.21) and low birth weight (PR=1.6; CI=1.14-2.28). Two deaths from congenital syphilis, one death from another cause and five stillbirths were reported.

**Conclusion:**

The results signify a long way until reaching the World Health Organization’s goal of eradicating congenital syphilis.

## Introduction

Worldwide estimates in 2012 indicated 927,936 maternal infections due to active syphilis and 350,915 adverse pregnancy outcomes^1^. This infection represents a serious public health problem, associated with perinatal complications such as congenital syphilis. Of the 350,915 adverse effects, 143,100 resulted in fetal deaths/stillborn, 61,860 neonatal deaths, 44,132 preterm/low-birth-weight infants and 101,813 infected infants^1^.

According to the Epidemiological Bulletin of Syphilis (2016), 33,365 cases of syphilis during pregnancy were reported in Brazil in 2015, reaching a detection rate of 11.2 syphilis cases in pregnant women per thousand live births. The rate in 2010 was 3.3 cases per thousand live births, showing an increase of 202% in five years. The data are even more worrying in the South and Southeast regions of the country; the detection rate was 15.1 and 12.6 syphilis cases in pregnant women per thousand live births, respectively, exceeding the national rate*. The number of notified congenital syphilis cases has also increased across the country*. In the last ten years, there has been a progressive increase in the incidence rate of congenital syphilis, from 2.0 cases per thousand live births in 2006 to 6.5 cases per thousand live births in 2015*. 

Congenital syphilis is most often associated with pregnant women who are not screened for syphilis, and/or those that are often not treated properly or even do not receive any treatment. According to the Brazilian Ministry of Health, 56.5% of pregnant women with syphilis received inadequate treatment, 27.3% did not receive any treatment, 12.1% of cases were ignored and only 4.1% received proper therapy*. It is worth mentioning that the majority of pregnant women who do not receive treatment or who are not treated properly can transmit the infection to their fetus, which can lead to fetal death, neonatal death, prematurity, low birth weight or congenital infection^2^ ^-^ ^3^. 

Despite the World Health Organization (WHO) launching the initiative to eliminate syphilis transmission^1^ in 2007, there has been an increase in infection during pregnancy in recent years^4^ ^-^ ^6^. In addition, few Brazilian studies have investigated the results of syphilis in pregnancy associated with maternal and perinatal factors^5^ ^-^ ^8^, with no studies being conducted in southern Brazil. 

In view of this scenario, the objective of this study was to analyze the prevalence of syphilis in during pregnancy and its association with socioeconomic characteristics, reproductive history, prenatal care and labor, and newborn characteristics.

## Method

A retrospective, cross-sectional study conducted according to the recommendations of STROBE (Strengthening the Reporting of Observational Studies in Epidemiology) with gestational and congenital syphilis reports of people residing in the 15^th^ Health Region of the State of Paraná from 2011 to 2015. 

The Brazilian Unified Health System (*SUS*) databases used in this study were: the “Information System for Notifiable Diseases” (*Sistema de Informação de Agravos de Notificação* - *SINAN* - Syphilis in pregnancy and Congenital syphilis); the “Live Births Information System” (*Sistema de Informação sobre Nascidos Vivos* - *Sinasc*); and the “Mortality Information System” (*Sistema de Informação sobre Mortalidade* - *SIM*).

*SINAN* was used to access the total number of syphilis reports in gestation and congenital syphilis for studied the period and region. *Sinasc* was used to obtain data on the obstetric history of the mother in cases of live births, in addition to newborn data. Lastly, the *SIM* database was used to obtain data regarding the obstetric history of mothers in cases of abortion and stillbirths, in addition to records of neonatal death due to congenital syphilis. 

A linkage of the *SINAN*-gestational syphilis, *Sinasc* and *SIM* databases was carried out using the variables: “patient’s name”, “date of birth and/or age” in the *SINAN*-gestational syphilis; and “mother’s name”, “maternal date of birth and/or age” in the *Sinasc* database; and the variable “mother’s name” in the *SIM* database. After unification of the databases, it was observed that 36 pregnant women who had been reported as having syphilis during pregnancy had no records of their babies in the *Sinasc* or *SIM* databases, and were excluded from the analysis. 

After performing the (records) linkage between syphilis in pregnancy and congenital syphilis in the databases, it was observed that 14 newborns did not have records of their respective mothers’ reports, therefore they were also excluded from the analysis. Another 15 cases were subsequently excluded for not having their respective records included in the *Sinasc* or in the *SIM* databases, therefore resulting in 147 cases of congenital syphilis included in the analysis.

A ratio of the total number of reported cases of syphilis during pregnancy (306) divided by the number of pregnancies in the period multiplied by 100 was used to estimate syphilis prevalence in gestation (number of existing cases of the disease in the population). The number of pregnancies was obtained by the sum of live births, abortions and stillbirths recorded in the period, while the number of reported cases divided by the number of live births multiplied by 1,000 was used for the detection rate of syphilis in pregnant women (annual incidence of the disease).

The incidence of congenital syphilis (number of new cases) corresponded to the total number of notified cases of congenital syphilis in children under one year of age, by the total number of live births of mothers living in the same location and in the same period, multiplied by 1,000. Absolute and relative frequencies as well as the prevalence ratio were calculated according to sociodemographic, reproductive variables, newborn characteristics and access to health services for syphilis cases during pregnancy reported during the study period. Fisher’s exact test was used for expected values below five. The analyzes were performed using SPSS software version 20.1.

The study was approved by the Standing Committee on Ethics in Research Involving Human Beings of the State University of Maringá, under the opinion number 1.246.907/15.

## Results

**Figure 1:**
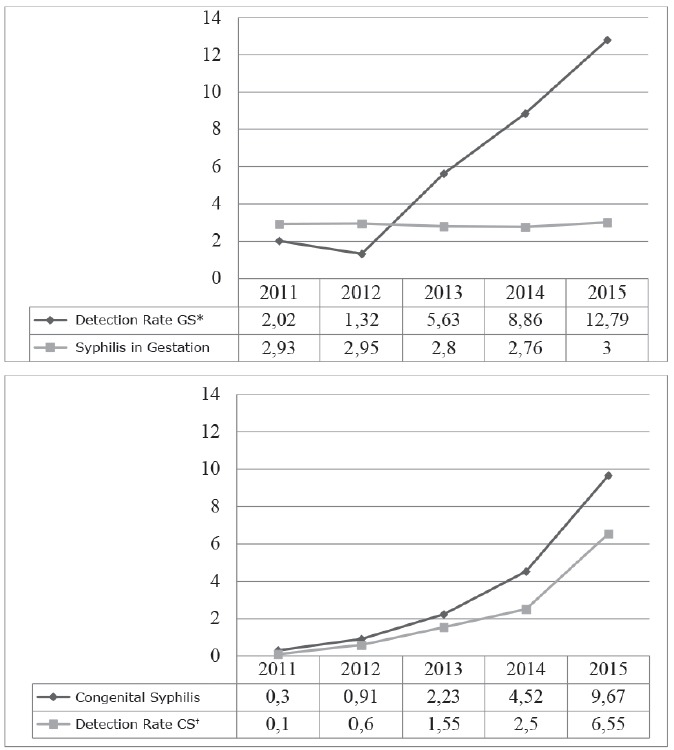
Prevalence and detection rate of gestational syphilis (A), Incidence and detection rate of congenital syphilis (B), according to the year. 15^th^ Health Region, Maringá, PR, Brazil, 2016

306 cases of syphilis in pregnancy were notified in the 15^th^ Health Region of the State of Paraná from the pregnancies occurring between 2011 and 2015, with a prevalence of 0.57%. A slight increase was observed in the prevalence of notified cases from 2.93% in 2011 to 3.00% in 2015. However, the detection rate increased considerably from 2.02 cases/thousand live births in 2011 to 12.79 cases/thousand live births in 2015 (Figure 1).

*GS - Gestational syphilis; ^†CS -^ Congenital Syphilis

There were 176 reported cases of congenital syphilis, with a progressive annual increase in the disease incidence from 0.30 cases/thousand live births in 2011 to 9.67 cases/thousand live births in 2015, and a detection rate in the same year of 6.55 cases/thousand live births (Figure 1).

Regarding the sociodemographic characteristics, 67.41% of the pregnant women were in the age group of 20 to 34 years, and 22.59% were adolescents (≤19 years), which was the age group with the highest infection prevalence in the gestational period compared to other ages (Table 1). 

**Table 1:** Prevalence ratio of reported syphilis cases during pregnancy according to sociodemographic, reproductive and access to health services characteristics. 15^th^ Health Region, Maringá, PR, Brazil, 2016

		N* (270)	%	PR^†^	CI^‡^ (95%)
Maternal age					
	≤ 19 years	61	22.59	1.7	1.30-2.31
	20 to 34 years	182	67.41	-	
	35 or older	27	10.00	0.8	1.27-0.56
Skin color/ethnicity					
	White	162	60.00	-	
	Non-white	105	38.89	4.6	3.62-5.76
	Ignored	3	1.11		
Maternal educational level					
	Illiterate	2	0.74	122.3	85.34-inf.^§^
	<8 years	86	31.85	15.4	12.60-18.86
	≥8 years	151	55.93		
	Ignored	31	11.48	-	
Occupation					
	Yes	74	27.41	-	
	No	151	55.93	4.5	3.50-5.83
	Student	13	4.81	4.6	2.69-7.90
	Ignored	32	11.85	4.3	2.91-4.49
Previous live births^║¶^					
	Yes	158	58.52	1.2	1.10-1.79
	No	112	41.48	-	
Previous Fetal loss/abortion^║¶^					
	Yes	60	22.22	1.7	1.27-2.24
	No	210	77.78	-	
Parity^§║^					
	Primiparous	95	35.19	-	
	Multiparous	175	64.81	1.9	1.45-2.37
Prenatal care during pregnancy**					
	Yes	138	93.88	-	
	No	6	4.08	7.4	3.68-14.97
	Ignored	3	2.04		
Prenatal consultations^¶^**					
	< 7	75	27.78	1.8	1.40-2.38
	≥ 7	195	72.22	-	
Type of delivery^║¶^					
	Cesarean section	154	57.04	-	
	Vaginal delivery	116	42.96	2.4	1.91-3.06

Source: Information System for Notifiable Diseases (*SINAN*) and *Live Births Information System* (*Sinasc*).

*N - Notifications of syphilis during pregnancy; †PR - Prevalence ratio.; ‡CI - Confidence interval; §Fisher’s Exact Test; ║Data extracted from the *Sinasc*; ¶Data extracted from the SIM; **Data extracted from the *SINAN* for congenital syphilis.

The occurrence of syphilis during pregnancy was associated with non-white skin color/ethnicity (PR=4.6, CI=3.63-5.6), educational level of less than eight years (PR=15.4, CI=12.60-18.86), absence of paid occupation (PR=4.5, CI=3.50-5.83) and being a student (PR=4.6, CI=2.69-7.90). Regarding reproductive history, there was an association of syphilis in gestation with multiparous women (PR=1.9, CI=1.45-2.37) and with a history of fetal loss (PR=1.7, CI=1.27-2.24) (Table 1). 

Table 1 also shows that the occurrence of syphilis is 7.4 times higher in women who did not receive prenatal care. Likewise, women who performed less than 7 prenatal consultations and those who had vaginal delivery had a higher prevalence of syphilis infection (PR=1.8 and 2.4, respectively).

Among the reported cases of gestational syphilis, 78.23% of pregnant women were diagnosed with the infection during prenatal care, 83.33% presented a reactive non-treponemal Venereal Disease Research Laboratory (VDRL) test, and 62.59% of the diagnoses were confirmed by the Fluorescent Treponemal Antibody-Absorption (FTA-Abs) non-treponemic test. At the time of delivery and in curettage cases, 74.83% of the women had a reactive VDRL test, and the FTA-Abs was positive in 41.50% of the cases. It is also worth mentioning that the infection was diagnosed in the first gestational trimester in 39.26% of the cases and in the second trimester in 31.11% of the cases, with the majority of notified cases classified as “primary” syphilis (61.11%) (Table 2).

**Table 2:** Diagnosis and clinical classification of maternal syphilis according to characteristics of prenatal care and treatment of pregnant women and their partners. 15^th^ Health Region, Maringá, PR, Brazil, 2016

		N*	%
Diagnosis of syphilis^†^			
	During prenatal care	115	78.23
	During delivery/curettage	12	8.16
	After delivery	14	9.52
	Not performed/Ignored	6	4.09
VDRL^‡^ during prenatal care			
	Reactive	225	83.33
	Not reactive	32	11.85
	Not performed/Ignored	13	4.82
FTA-Abs^§^ during prenatal care			
	Reactive	169	62.59
	Not reactive	42	15.56
	Not performed/Ignored	59	21.85
VDRL^‡^ during delivery/curettage^†^			
	Reactive	110	74.83
	Not reactive	25	17.01
	Not performed/Ignored	12	8.16
FTA-Abs^§^ during delivery/curettage^†^			
	Reactive	61	41.50
	Not reactive	15	10.20
	Not performed/Ignored	71	48.30
Gestational trimester			
	First	106	39.26
	Second	84	31.11
	Third	71	26.30
	Ignored	9	3.33
Clinical classification			
	Primary	165	61.11
	Secondary	25	9.26
	Tertiary	14	5.19
	Latent	21	7.78
	Ignored	45	16.67
Treatment of the pregnant women			
	Adequate	125	46.30
	Inadequate/Not performed	145	53.70
Treatment of the partner			
	Yes	97	35.93
	No/Ignored	173	64.07
Reason for not treating the partner^║^			
	He no longer has contact with the pregnant woman	43	15.93
	He was not notified/invited for treatment	15	5.56
	He was notified/invited, but he did not show up	22	8.15
	He refused treatment	5	1.85
	Non-reactive serology	28	10.37
	Another reason	78	28.89
	Ignored	79	29.26

Source: Information System for Notifiable Diseases (*SINAN*) and Live Births Information System (*Sinasc*).

*N - Notifications of syphilis during pregnancy; †Data extracted from the Sinan for congenital syphilis; ‡VDRL *-* Venereal Disease Research Laboratory; § FTA-Abs Fluorescent Treponemal Antibody-Absorption; ║Only for cases of partners of pregnant women who did not receive treatment.

The treatment of pregnant women with syphilis was considered inappropriate or was not performed in 53.70% of the cases. There was no treatment for the partner of the pregnant woman in 64.07% of the cases, and the most reported reasons were: the pregnant woman lost contact with the partner, the partner’s serology was not reactive, the partner was invited but he did not attend, among other reasons (Table 2).

The newborns’ characteristics which were associated with the occurrence of syphilis were gestational age less than 36 weeks (PR=1.6, CI=1.17-2.21) and birth weight below 2500g (PR=1.6, CI=1.14-2.28) (Table 3). 

**Table 3:** Prevalence ratio of newborn characteristics from mothers who had been notified of having syphilis during pregnancy. 15^th^ Health Region, Maringá, PR, Brazil, 2016

Newborn characteristics		N*	%	PR^†^	CI^‡^ (95%)
Gender^§║^					
	Female	131	48.52	-	
	Male	139	51.48	1.0	0.80-1.29
Gestational age^§║^					
	< 37 weeks	46	17.04	1.6	1.17-2.21
	≥ 37 weeks	224	82.96	-	
Birth weight^§║^					
	< 2,500 g	37	13.70	1.6	1.14-2.28
	≥ 2,500 g	233	86.30	-	
Apgar 1^║^					
	< 7	21	7.87	1.2	0.80-1.94
	≥ 7	246	92.13	-	
Apgar 5^║^					
	< 7	3	1.12	1.2	0.37-3.63^¶^
	≥ 7	264	98.88	-	
Malformation^§║^					
	Yes	3	1.12	1.4	0.45-4.37^¶^
	No	264	98.88	-	

Source: Information System for Notifiable Diseases *(SINAN)* and Live Births Information System *(Sinasc).*

*Newborns of mothers notified with syphilis during pregnancy; †PR - Prevalence ratio; ‡CI - Confidence interval; §Data extracted from the *Sinasc*; ^║^Data extracted from the *SIM*; ¶Fisher’s Exact Test;

Regarding the care characteristics of newborns with congenital syphilis, the gestational age at diagnosis was less than two days in 80.27% of the cases, 53.74% of the serology for VDRL were reactive, and 14.29% were not performed. Moreover, the VDRL for the cerebrospinal fluid of 34.69% of newborns was not collected, while long-bone X-rays did not present any alteration in 42.18% of the cases and were not performed in 28.57% (Table 4). 

**Table 4:** Diagnosis and treatment of newborns notified with congenital syphilis. 15^th^ Health Region, Maringá, PR, Brazil, 2016

Characteristics of newborn care	N* (147)	%
Age at diagnosis (days)^†^		
< 2	118	80.27
2 to 28	21	14.29
≥ 28	8	5.44
Results from the VDRL^‡^ of peripheral blood^†^		
Reactive	79	53.74
Not reactive	39	26.53
Not performed	21	14.29
Ignored	8	5.44
Results from the VDRL^‡^ of the cerebrospinal fluid^†^		
Reactive	6	4.08
Not reactive	52	35.37
Not performed	51	34.69
Ignored	38	25.85
Alterations in long-bone X-ray^†^		
Yes	5	3.40
No	62	42.18
Not performed	42	28.57
Ignored	38	25.85
Case evolution^†^		
Alive	131	89.12
Death by congenital syphilis	2	1.36
Death due to other causes	1	0.68
Stillborn	5	3.40
Ignored	8	5.44
Treatment plan^†^		
Crystalline Penicillin G 100,000 to 150,000 Ul/kg/day	27	18.37
Procaine Penicillin G 50,000 Ul/kg/day	3	2.04
Benzathine Penicillin G 50,000 Ul/kg/day	5	3.40
Other plan	26	17.69
Not performed	70	47.62
Ignored	16	10.88

*N - Newborns notified with congenital syphilis; †Data extracted from the *SINAN* for congenital syphilis; ‡ VDRL *-* Venereal Disease Research Laboratory;

Regarding treatment, Crystalline Penicillin G was prescribed for 18.37% of the newborns, and no therapeutic regimen was performed in 47.62% of the cases. Regarding the evolution of the case, two newborns (1.36%) died from congenital syphilis and five (3.40%) were stillborn. 

## Discussion

To the best of our knowledge, this is the first study conducted in southern Brazil that investigated the results of syphilis during pregnancy associated with maternal and perinatal factors. The present study found a detection rate of syphilis during gestation of 12.79 cases/thousand live births, which is similar data to that found in the Southeast region (12.6), and above the national rate (11.2). We also found an association of non-white skin color/ethnicity, low educational level, and companion absence during prenatal care with syphilis during pregnancy, as well as the occurrence of prematurity and low birth weight associated with gestational syphilis. 

The incidence rate of congenital syphilis in the studied region for the year 2015 was 9.67 cases/thousand live births; higher than the national incidence in the same year, which was 6.5 cases per thousand live births, and far from the stipulated target of 0.5 established by the “Strategy and Plan for Eliminating the Vertical Transmission of HIV and Congenital Syphilis - *Estratégia e Plano de Eliminação da Transmissão Vertical do HIV e da Sífilis Congênita*”* ^,^ **. Further studies should be performed to elucidate the causes of syphilis rates in gestation and congenital disease being higher than the national average, considering that this is a region with a high human development index and prenatal coverage.

These figures are high and the results are even more worrying considering that these numbers may be underestimated, as notification in Brazil reaches only 32% for syphilis cases in the gestational period, and 17.4% for congenital syphilis^6^. Without notification of suspected cases, there is no adequate investigation and treatment for either the pregnant woman or the baby, thus increasing the cases of events resulting from the disease. Investing in epidemiological surveillance is the first step in controlling the reemergence of syphilis.

In this study, pregnant women younger than 20 years were at a higher risk of contracting the infection during pregnancy. This can be explained by the vulnerability of the adolescent population, which is more exposed to sexually transmitted diseases as this phase corresponds to an emotional, cognitive and age immaturity, in addition to being a period of discoveries and great influence by social groups**. A study carried out with 90 adolescents aged 14 to 16 years covering the public and private networks on the perception about sexual practice, found that sexual intercourse among adolescents is increasingly precocious and accompanied by negligence regarding the use of contraceptives, both to prevent unplanned pregnancy and to prevent Sexually Transmitted Diseases (STDs)^9^.

Characteristics such as non-white skin color/ethnicity, low educational level and absence of paid occupation are variables that were statistically associated with gestational syphilis, and are similar to other studies*^5^ ^,^ ^8^ ^,^ ^10^ ^-^ ^11^. This is often the profile of individuals with a less favorable socioeconomic condition and with less access to quality healthcare. However, it cannot be said that syphilis is exclusively a risk condition for the most deprived populations; on the contrary, anyone can acquire the infection regardless of social or economic condition, however the risk is higher in more vulnerable populations^12^.

Multiparous women with a history of fetal loss and without prenatal care or with a low number of prenatal visits were also statistically associated with the occurrence of syphilis during gestation. In Brazil, prenatal coverage is greater than 95%^13^ ^-^ ^15^. However, it is known that high prenatal coverage rates do not necessarily mean quality and adequacy of care. There are several factors that produce adequate prenatal care, such as gestational age at the beginning of prenatal care, number of consultations, and the performance of routine examinations, among others^16^. 

In addition, there are some pregnant women without any prenatal follow-up or prenatal consultations; these women constitute a socially vulnerable population and manifest a higher prevalence of syphilis during pregnancy^13^ ^-^ ^15^.

Regarding the type of delivery, syphilis was more prevalent in women who had their children through vaginal delivery. According to some authors, this data may be related to the socioeconomic condition^16^. In Brazil, the highest cesarean rates are historically related to factors such as more privileged socioeconomic situation, having white skin color/ethnicity, having a higher educational level and access to private health services, while vaginal delivery is still more common in public health services in women of lower socioeconomic power, and with lower education levels^17^ ^-^ ^18^.

With regard to the early diagnosis of syphilis in pregnant women, the majority of women were diagnosed during prenatal care. Some studies show that outcomes of non-identification and (lack of) early treatment of infection during pregnancy are severe for the infant, and these outcomes depend on the stage of maternal infection and the gestational age of fetal exposure, which may lead to prematurity, abortion, stillbirth and neonatal death^14^ ^,^ ^19^ ^-^ ^20^. In addition, quality prenatal care with early adherence by the pregnant woman to actions for health promotion, sexual guidance and reproductive orientation, as well as accomplishing the protocol of examinations recommended during the gestational period is essential for preventing harm to the baby*. 

In addition, the majority of people with syphilis tend to be unaware of the infection and can transmit it to their sexual partner(s), and in the case of gestation to the fetus, causing severe consequences. This is due to an absence or lack of symptoms depending on the infection stage**. It is essential that pregnant women are examined by trained professionals and screened for syphilis regularly in order to detect any clinical or serological signs of infection^14^ ^-^ ^21^.

In the present study, almost all pregnant women reported having syphilis were screened using VDRL during prenatal care, with the majority of them presenting reactive serology. The treponemal test (FTA-Abs) responsible for confirming the diagnosis was not performed in all pregnant women; however, most serologies among those who performed it were reactive. Syphilis diagnosis is basically serological, hence the importance of all pregnant women being tested at the first prenatal visit in the first trimester of pregnancy, and they should repeat the test at the beginning of the third trimester around 28 weeks, so that appropriate therapy is instituted in a timely manner if necessary^16^ ^-^ ^22^.

Non-treponemal tests such as VDRL can produce false-positive results, and therefore must be confirmed by treponemal tests which are more specific. Numerous conditions can lead to positive results for syphilis in non-treponemal tests; pregnancy itself is a frequent cause of false positive results for syphilis, however with low titration^22^.

Most non-treponemic serologies were reactive during delivery or curettage and the confirmation test (FTA-Abs) was positive in most pregnant women. This result may reflect possible reinfection of the pregnant woman. 

Elevated titers in non-treponemal tests in relation to the previous examination points to reinfection, and a new treatment should be initiated**. It is also important to consider that there is high risk of reinfection even if women are treated appropriately according to the clinical stage, but their partners are not; these situations ratify the importance of follow-up for the pregnant woman after treatment^21^ ^,^ ^23^ ^-^ ^24^.

It is important that opportunities to prevent syphilis are not lost. If confronted with a sign and clinical symptom and/or non-treponemic positive serology, and in the impossibility of confirming the diagnosis, the conduct should be to immediately treat the pregnant woman and advise her to notify her partner to perform the treatment, thus avoiding reinfection of the woman^7^ ^-^ ^8^ ** ^.^ The treatment should be performed in the unit where the diagnosis was performed, not requiring hospitalization**.

The treatment recommended by the Brazilian Ministry of Health and the World Health Organization is the intramuscular use of benzathine penicillin G with a therapeutic regimen according to the clinical classification of the infection** ^19^ ^,^ ^25^. In the gestational period, penicillin G benzathine is the only effective medication against vertical transmission and for treating congenital syphilis** ^19^ ^,^ ^25^. It is important to emphasize that the treatment is not only the medication, as some other criteria need to be considered in order for the medication to be effective which are according to the recommended regimen/plan for the disease phase, commencing the treatment up to 30 days before delivery, and treatment of the partner**.

In this study, it was observed that the partner was not treated and the main reasons were absence of contact with the pregnant woman, unreactive serology and treatment refusal. Other studies also address the importance of treating the partner(s) in curing gestational syphilis and preventing vertical transmission^5^ ^,^ ^7^ ^-^ ^8^, thus not only indicating the importance of health education for pregnant women, but also for the sexual partners.

According to the clinical classification of syphilis, in most cases the infection was classified as primary, which is the first clinical stage of the disease. A historical series of syphilis cases in pregnant women and congenital syphilis carried out in Brazil from 2005 to 2016 also found that the majority of cases were classified as primary syphilis; however, they indicated the possibility of inadequate classification*. It is important to emphasize that in the impossibility of establishing the clinical evolution of the disease, the appropriate classification is “latent syphilis of unknown duration”, since the treatment for primary syphilis would be insufficient in cases where it is not the clinical classification of the disease*.

Even after treatment, non-treponemal tests (VDRL) need to be performed in pregnant women with monthly frequency for cure control. Titer reduction of around two dilutions in three months and three dilutions at six months after treatment is an indication of success in therapy. The persistence of low titers is called a serologic scar and can last for years or even a lifetime. A new treatment should be considered in cases of new exposure**.

Regarding the possible perinatal outcomes caused by the occurrence of syphilis in the gestational period, this study revealed that fetal or neonatal death, low birth weight, prematurity, and other malformations due to congenital infection** ^3^ ^,^ ^10^ ^-^ ^11^ ^,^ ^26^ were positively associated with maternal infection. In a systematic review and meta-analysis, the authors found an association between the aforementioned characteristics and gestational syphilis^26^. Similarly, a multicenter study of maternal syphilis morbidity and adverse events associated with gestation in India, Nigeria and Zambia also related prematurity, low birth weight, and other outcomes such as stillbirth and death to the occurrence of syphilis during pregnancy^27^. 

In relation to newborns reported as having congenital syphilis, they were diagnosed at less than two days of life; data that corroborate those presented by the Epidemiological Bulletin, in which 96.4% of cases were diagnosed in the first weeks of life*. Early diagnosis allows greater success in treatment and avoiding late complications such as: “Sabre-like tibial deformity”/Saber shin, prominence of the forehead (Olympian brow), or neurological deafness, among others**.

A high frequency of not performing long-bone X- rays and a high rate of non-VDRL collection from the cerebrospinal fluid were also observed. These exams are part of the care protocol for newborns of mothers with a history of syphilis during pregnancy, and are important for the diagnosis of syphilis-related alterations. The low/under performance of these tests has also been reported by other authors** ^28^.

There are several outcomes for the newborns of mothers with untreated or inadequately-treated syphilis. In the present study, two deaths were found for congenital syphilis, one death for another cause and five stillbirths, thus corroborating findings by other studies^10^ ^-^ ^11^ ^,^ ^26^. Even if they do not appear quantitatively significant, these outcomes are important when considering that these are preventable events by quality maternal and child care. 

It should be noted that this study has some limitations such as the use of secondary data, since they are conditioned to the quality of the records, in addition to allowing for estimating how much the frequency of underreporting can distort the results, which can even lead to regional disparities. However, despite their limitations, the databases used are considered reliable, of good quality and with reliable information. In addition, the linkage of different databases enabled a more comprehensive analysis of the reported syphilis cases during pregnancy.

## Conclusion

The results of this study show that there is still much progress to be made towards the WHO goal of eliminating congenital syphilis as a public health problem. The prevalence of gestational syphilis was 0.57%, and the analyzes indicated several variables associated with its occurrence such as age, non-white skin color/ethnicity, low education level and absence of prenatal follow-up. Prematurity and low birth weight were associated with gestational syphilis and were related to perinatal outcomes such as the occurrence of two deaths by congenital syphilis and five cases of stillbirth among the newborns of mothers with syphilis during pregnancy.

In order to reduce the prevalence of syphilis in pregnancy and congenital syphilis, it is essential that health professionals and the community become aware of the importance of early diagnosis and the effective treatment of women and their partners. The multiprofessional team is responsible for screening pregnant women in prenatal consultations, actions to raise awareness about the risks of unsafe sexual practices and the importance of self-care, especially among the most vulnerable populations. 

## Footnotes

* Ministry of Health/Ministério da Saúde (BR). Epidemiological Report: Syphilis/Sífilis [Internet]. Brasília: Ministério da Saúde; 2016. [Access Oct. 26th, 2016]. Available at: http://www.aids.gov.br/publicacao/2016/boletim-epidemiologico-de-sifilis

## Footnotes

** Ministry of Health/Ministério da Saúde (BR). Clinical protocol and therapeutic guidelines for comprehensive care for people with sexually transmitted infections (Protocolo clínico e diretrizes terapêuticas para atenção integral às pessoas com infecções sexualmente transmissíveis) [Internet]. Brasília: Ministério da Saúde; 2015c. [Access June 18th, 2016]. Available at: http://www.aids.gov.br/publicacao/2015/protocolo-clinico-ediretrizes-terapeuticas-para-atencao-integral-pessoas-com-infecc
